# Temperature Measurement Method for Blast Furnace Molten Iron Based on Infrared Thermography and Temperature Reduction Model

**DOI:** 10.3390/s18113792

**Published:** 2018-11-06

**Authors:** Dong Pan, Zhaohui Jiang, Zhipeng Chen, Weihua Gui, Yongfang Xie, Chunhua Yang

**Affiliations:** School of Information Science and Engineering, Central South University, Changsha 410083, China; pandong@csu.edu.cn (D.P.); shling@csu.edu.cn (Z.C.); gwh@csu.edu.cn (W.G.); yfxie@csu.edu.cn (Y.X.); ychh@csu.edu.cn (C.Y.)

**Keywords:** blast furnace, molten iron temperature, infrared thermography, skimmer, taphole, temperature reduction model

## Abstract

The temperature measurement of blast furnace (BF) molten iron is a mandatory requirement in the ironmaking process, and the molten iron temperature is significant in estimating the molten iron quality and control blast furnace condition. However, it is not easy to realize real-time measurement of molten iron temperature because of the harsh environment in the blast furnace casthouse and the high-temperature characteristics of molten iron. To achieve continuous detection of the molten iron temperature of the blast furnace, this paper proposes a temperature measurement method based on infrared thermography and a temperature reduction model. Firstly, an infrared thermal imager is applied to capture the infrared thermal image of the molten iron flow after the skimmer. Then, based on the temperature distribution of the molten iron flow region, a temperature mapping model is established to measure the molten iron temperature after the skimmer. Finally, a temperature reduction model is developed to describe the relationship between the molten iron temperature at the taphole and skimmer, and the molten iron temperature at the taphole is calculated according to the temperature reduction model and the molten iron temperature after the skimmer. Industrial experiment results illustrate that the proposed method can achieve simultaneous measurement of molten iron temperature at the skimmer and taphole and provide reliable temperature data for regulating the blast furnace.

## 1. Introduction

Blast furnace (BF) ironmaking, which refers to the continuous production of molten iron in BFs using coke, iron ore and flux, is one of the key processes in the iron and steel industry, and the molten iron temperature (MIT) produced by BF is a main parameter that reflects the BF condition, the molten iron quality, and the energy consumption status [1,2,3]. Therefore, a real-time measurement method of MIT would be of great use for maintaining a stable operation of a BF and regulating the molten iron quality [4,5].

During the tapping process, the molten iron inside the BF hearth flows through the taphole into the main pipeline and is transported to the steelmaking workshop after separating the slag and molten iron. A BF can be considered as a closed high-temperature large vertical reactor, as a result, there is no method to directly detect the MIT in the BF hearth. Considering that the taphole is connected to the blast furnace hearth, the MIT at taphole can be seen as the MIT in the hearth of the BF. However, given the high-temperature and harsh environment during the tapping process, direct detection of the MIT at the taphole of the BF is full of challenges, and the MIT after the skimmer is usually measured to reflect the MIT in the BF hearth [6,7].

Currently, the devices used to measure the MIT after skimmer can be classified into two types according to whether or not the device is in contact with molten iron: contact-type and non-contact-type. Contact-type devices include thermocouples, blackbody cavities, and so on [8,9,10]. The temperature detected by thermocouples is stable and accurate, but this detection approach is intermittent and loses a thermocouple in each measurement. In addition, BF operators are faced with a certain risk when measuring molten iron temperature near the main pipeline. The blackbody cavity is a sensor of the cavity-based blackbody, and it entails direct insertion of a temperature probe into molten iron and converts the infrared signal from the probe into a temperature signal, which enables continuous temperature measurement. However, the device has a short service life and high cost owing to the limitation of its material. Non-contact-type devices include infrared thermometers, colorimetric thermometers and infrared thermal imagers [11,12,13,14,15,16]. Infrared thermometry enables safe and continuous temperature measurement. An infrared thermometer can receive the infrared radiation of the measured object and convert the radiation into a voltage signal corresponding to the object’s temperature; a colorimetric thermometer can measure the temperature of the object according to the ratio of the two kinds of infrared radiation that are emitted by the object from two adjacent infrared bands. However, the measurement result of the infrared thermometer and colorimetric thermometer is an average temperature of a point, which is prone to be affected by environment. When there is an offset between the measured object and the infrared measurement instrument, there will be errors in the temperature measurement results, or even wrong results. Infrared thermal imagers can provide two-dimensional infrared images of the measured object [17,18]. Besides, these have no harmful radiation effect. If the measured object is in the field of view of the infrared thermal imager, whether the object is static or dynamic, its surface temperature information must be contained in the infrared thermal image, and we can obtain the object’s surface temperature through certain methods. Hence, the infrared thermal imager is better than the infrared thermometer and the colorimetric thermometer. 

At present, there is extensive literature regarding the application of infrared thermography for thermal and temperature measurement in many research areas. In [19], an infrared camera was used to realize the temperature measurement at the input and output of industrial continuous furnaces. Manara applied a long wavelength infrared radiation thermometer to measure the surface temperature of thermal barrier coatings during the operation of gas turbine engines [20]. Imaz designed an infrared thermometry system to measure the temperature of domestic induction heating cookers [21]. Feng analyzed the heat conduction performance of single-walled carbon nanotube thin films and measured the thermal conductance by infrared thermography [22]. In [23], Tanda used infrared thermography to detect and investigate the skin temperature response to physical activity. Yousefi adopted an infrared thermal imager to find a radiology gown with suitable thermal properties and track human body thermal variations during examination to prevent burning [24]. In consideration of the advantages of an infrared thermal imager, this paper adopts an infrared thermal imager to realize continuous and real-time measurement of the MIT.

To solve the difficulty of direct detection of the MIT at the taphole and avoid the harsh environment, a measurement method of the MIT is proposed based on infrared thermography and a temperature reduction model. Firstly, an infrared thermal imager is used to capture the infrared image of molten iron after the skimmer whose surrounding environment is not harsh. Then, the MIT after the skimmer is detected in real time by analyzing the temperature distribution of the molten iron flow. Finally, a temperature reduction model of the molten iron in the main pipeline is established, and the MIT at the taphole can be obtained continuously according to the model and the MIT after the skimmer.

The rest of this paper is organized as follows: Section 2 describes the proposed method for the measurement of MIT. Section 3 discusses the experimental results. Finally, Section 4 presents the conclusions.

## 2. Temperature Measurement Method

**Figure 1:**
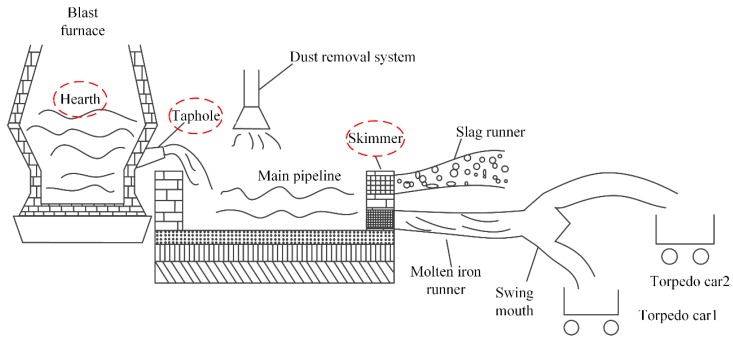
A typical blast furnace (BF) casthouse in ironmaking plants.

**Figure 2:**
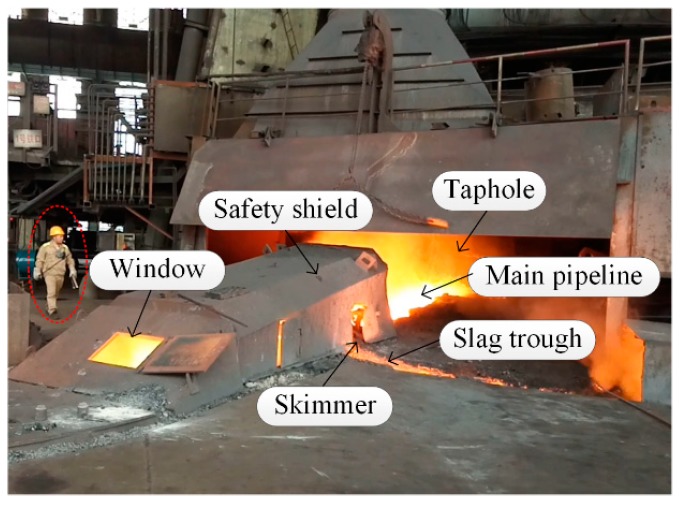
Real casthouse of No. 2 BF in an ironmaking plant.

A typical BF casthouse in ironmaking plants is shown in Figure 1. When the molten iron in the BF needs to be discharged, the taphole is opened by a machine, and the molten iron in the BF hearth flows into the main pipeline and is separated from slag at the skimmer. Figure 2 is the real casthouse of No. 2 BF in an ironmaking plant of Liuzhou Steel Co. Ltd (Liuzhou, China). in which an operator was preparing to measure the MIT after the skimmer with a thermocouple.

**Figure 3:**
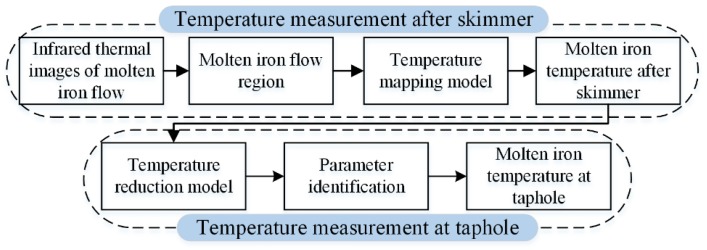
Summary of the temperature measurement method.

The main purpose of the proposed method is to achieve continuous temperature measurement of molten iron and reduce manual operation. Figure 3 is a brief summary of the proposed measurement method. Firstly, based on infrared thermography, the MIT after the skimmer is measured continuously. Then, the MIT at the taphole is calculated based on a temperature reduction model. The details are described below.

### 2.1. Testing Parameters of the Infrared Thermal Imager

In the BF casthouse, the environmental disturbances after the skimmer are much less than those at the taphole. Thus, in order to avoid the influence of environmental disturbances on infrared measurement results, these infrared thermal images of molten iron flow are captured by an infrared thermal imager after the skimmer.

The specific characteristic parameters of the infrared thermal imager used for molten iron temperature measurement are shown in Table 1. As shown in Table 1, the field of view of the infrared camera is 32.4° × 24.7° which is fixed. The horizontal distance between the window of the safety shield and the infrared camera is around 2 m, and the vertical installation height of the infrared camera is around 1.7 m.

When using the infrared camera for measurement, it is true that a smaller area can be visualized with a better spatial resolution by reducing the measurement distance between the infrared camera and the measured object. However, the MIT is around 1500 °C, and thus its emits strong infrared radiation. If the infrared thermal imager is installed too close to the molten iron, the body temperature of the infrared camera will rise due to high temperature radiation. According to Table 1, we know that the infrared camera’s operating temperature is between −25 °C and 55 °C. Taking the infrared camera’s operating temperature into consideration, it is necessary for there to be a certain distance between the infrared camera and the molten iron flow. Thus, there is a contradiction between spatial resolution and temperature measurement distance. The distance is not as long as possible, and it is not as close as possible. We need to compromise to choose the proper measurement distance. In order to obtain the proper measurement distance, we used a thermometer to measure the atmospheric temperature in front of the safety shield’s window, and found that the atmospheric temperature 1.5 m from the front of the window was about 55 °C. Hence, the measurement distance must be larger than 1.5 m to avoid long-term operation of the infrared camera in the high temperature state. In other words, 1.5 m is the minimum safe distance for the infrared thermal imager.

According to on-site research, we know that both the width and length of the safety shield’s window are 0.5 m and the inclination of the window is about 50°. The installation height of the infrared camera is preferably 1.7 m for the convenience of blast furnace operators. Because it is best to make the lens of the infrared camera directly face the window, the horizontal distance between the camera and the window can be 2 m which also satisfies the requirement of safe distance.

### 2.2. Temperature Measurement of Molten Iron after Skimmer

#### 2.2.1. Determination of Molten Iron Flow Region

**Figure 4:**
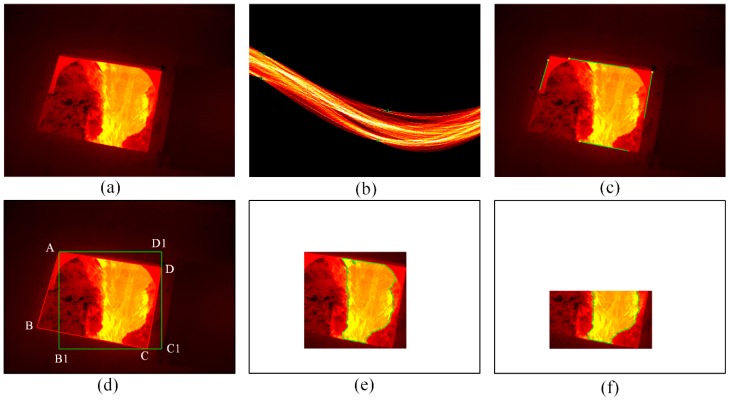
Determination of the region of interest (ROI). (**a**) Infrared thermal image of molten iron flow; (**b**) Hough transform; (**c**) results of Hough transform; (**d**) rectangle in the window; (**e**) edges after binarization, morphological operation; (**f**) ROI to be analyzed.

Figure 4a shows the captured infrared thermal image of molten iron flow after the skimmer. It is easy to know that there is molten iron, an oxide layer, trench wall and slag heap in the image. Since infrared thermal images are captured through the window of safety shield, the molten iron flow region that we are interested in only accounts for part of the infrared thermal image.

Generally, the portion of an image that we are particular interested in can be referred to as the region of interest (ROI). In this case, we define the rectangle region that contains molten iron flow as the ROI1 and the molten iron flow as ROI2. After defining the ROI, it is unnecessary to process the whole image, which leads to a reduction in computation time. Hence, we proposed a method to determine the ROI automatically. The method consists of two steps: the first step is to determine the ROI1 that contains all temperature information in ROI2, and the second one is to detect the ROI2 in the ROI1.

Step 1: Determining the region of interest (ROI1)

Since the molten iron flow only occupies part of the infrared thermal image, it is unnecessary to process the whole image. In order to reduce the computation cost, it is necessary to reduce the image area to be processed and only deal with the molten iron flow region.

The molten iron flow is captured though the window of the safety shield, and it is located inside the window in the infrared image. Thus, the determination of the ROI1 begins with the location of the window. It is obvious that the window’s edges are straight lines. Considering the advantages of the Hough transform in detecting straight lines, the Hough transform is applied to determine the four straight lines representing the window’s edges [25], and the transform results are shown in Figure 4b in which the four highlighted points correspond to the four lines shown in Figure 4c. Then, based on the location information of four lines in Figure 4c, we can obtain the equations of the four lines by linear fitting. Furthermore, the coordinates of four end points of the window can be obtained by calculating the four intersections of these lines, and the four end points are denoted as A, B, C and D respectively, as shown in Figure 4d.

Finally, the coordinates of A, C and D are used to determine a rectangle on the premise that the rectangle contains all the information of molten iron flow, and the rectangle AB1C1D1, which is the ROI1, is shown in Figure 4d. Compared with the whole image, the area of the image to be processed is reduced by 75% and all the molten iron flow information is retained. The following operation is performed on the ROI1.

Step 2: Detecting the ROI2

After determining the ROI1, several image processing techniques are applied to obtain the ROI2. Firstly, the ROI1 is processed by binarization in which the threshold is set as 0.4 to highlight the edges of molten iron flow. Then, morphological operations, which include dilation and erosion, are adopted to process the binarized image [26]. Dilation and erosion are used to eliminate the regions that are wrongly classified as molten iron flow. Next, the Canny operator, which has a better performance in the existing edge detection operators, is used to detect the edges of molten iron flow [27]. Finally, considering the molten iron does not have perfect borders and there may be noise in the detected edges, the resulting edge map is processed by open operation and skeletonization. Open operation is performed to remove small areas, and the purpose of skeletonization is to obtain the edges that are made up of individual pixels. The boundaries of molten iron flow, which are marked in green, are shown in Figure 4e, and the region determined by the boundaries is known as the ROI2.

In the ROI2, the oxidation reaction between the molten iron and air results in the formation of oxide films on the surface of the molten iron [28,29]. According to Plank’s law, the infrared radiation has an intrinsic link with the object’s surface temperature, and the higher the surface temperature, the greater the intensity of the infrared radiation, and the brighter the object on the infrared thermal imager. The infrared thermal imager can receive the infrared radiation from the object, and convert it into a temperature value. The infrared thermal images captured after the skimmer provide the temperature information of the molten iron flow. Since the actual temperature of the oxide film is much lower than the actual molten iron temperature, as a result, the brightness of the oxide film is not as high as that of molten iron in the infrared thermal image. From Figure 4e, it can be seen that most of the oxide film is distributed in the middle of the molten iron flow’s surface, and the unoxidized molten iron is distributed on both sides of the molten iron flow.

#### 2.2.2. Temperature Mapping Model

After determining the ROI2, a temperature mapping model is developed to map the temperature data in the ROI2 to the MIT. In order to obtain the temperature data in the ROI2, a matrix operator that consists of zero and one is defined as follows according to the position of pixels in the boundaries of the ROI2.

(1) $$B_{o}\left(i,j\right)=\left\{ \begin{array}{ll} 1, & if\;T(i,j)\;is\;in\;\mathrm{ROI}2\\ 0, & otherwise \end{array}\right.$$

where $B_{o}\left(i,j\right)$ and T(i,j) denote the element in the matrix operator and the temperature value corresponding to the pixel at abscissa i and ordinate j in the ROI2, respectively.

We use $T_{ROI}$ and $T_{r}$ denote the temperature data in the ROI2 and the ROI1, respectively. Then, the $T_{ROI}$ can be expressed as the dot product of matrix $B_{o}$ and matrix $T_{r}$. By preserving non-zero elements in the matrix $T_{ROI}$, we can obtain the temperature data corresponding to the ROI2.

(2) $$T_{ROI}=B_{o}\cdot T_{r}$$

For molten iron, it flows from the taphole to the skimmer, and then into the torpedo car after the skimmer during the tapping process. When the infrared camera is fixed at the installation position, the molten iron flow is always in the field of view of the infrared camera, and it is the molten iron flow in the field of view that are processed to obtain the molten iron temperature after skimmer. Besides, the position and state of the molten iron flow after the skimmer is relatively stable during a stable tapping process. Therefore, when the ROI2 changes in different infrared thermal images, we can repeat the image processing techniques without changing the parameters in these techniques to update the operator $B_{o}$, and obtain the temperature information in the ROI2.

There is molten iron and oxide film in the ROI2, and their temperatures are different; thus, it is possible to distinguish the molten iron from the oxide film from their temperature distribution. The oxide film accounts for the majority of the ROI2, and as a result the distribution shape of molten iron may be overshadowed by the distribution of oxide film. Considering most of the molten iron is distributed in the lower part of the ROI2, a proportionality coefficient, which ranges from 0 to 1 and determines which part of the ROI2 to use to obtain the temperature distribution, can be set to highlight the distribution shape of molten iron. Figure 4f shows the ROI2 to be analyzed when the coefficient is set to 0.67. The temperature mapping model is on the basis of histogram processing [30].

**Figure 5:**
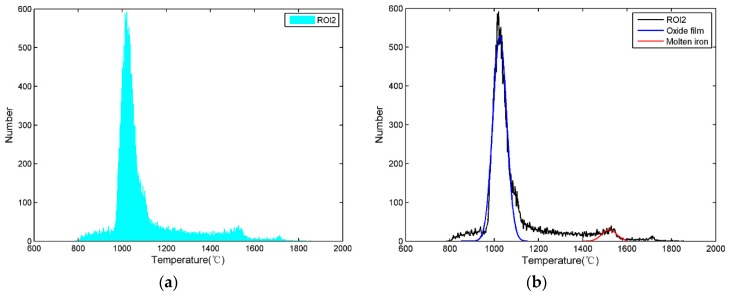
Temperature distribution histogram of the ROI2. (**a**) Histogram of the ROI2; (**b**) fitted curve of the ROI2.

By counting the number of different temperature data in the ROI2, the temperature distribution histogram of the ROI2 can be obtained, which is shown in Figure 5a. Since the ROI2 is mainly composed of molten iron and oxide film, there are two peaks corresponding to these two materials in Figure 5a. Because the oxide film accounts for most of the ROI2 while the molten iron accounts for a small portion, the high peak on the left corresponds to the oxide film and the low peak on the right corresponds to molten iron. The emissivity of molten iron and oxide film is listed in Table 2, and we know that the emissivity of the oxide film is higher than that of molten iron. Since the set emissivity of the infrared thermal imager is the molten iron emissivity, if the real temperature of the molten iron and the real temperature of the oxide film are equal, the ability of the oxide film to emit infrared radiation is stronger than that of molten iron, and the displayed oxide film temperature on the infrared thermal image is higher than the displayed molten iron according to the principle of infrared thermography. However, because of the radiant heat dissipation and the convection heat dissipation of the oxide film, the real oxide film temperature is indeed lower than that of molten iron. Although the ability of the oxide film to emit infrared radiation is stronger than that of molten iron, the displayed oxide film temperature on the infrared thermal image is lower than that of the molten iron.

In general, according to the temperature difference between the oxide film and the molten iron and the distribution characteristics of the temperature data, it is known that the peak on the left represents the oxide film temperature and the peak on the right represents the molten iron temperature in Figure 5a. The distribution of molten iron and oxide film approximately satisfies Gaussian distribution, so Gaussian functions are utilized to fit the peaks of molten iron and oxide film.

(3) $$f_{iron}\left(T\right)=a_{iron}\exp\left[-{\left(T-T_{iron}\right)}^{2}/b_{iron}^{2}\right]$$

(4) $$f_{film}\left(T\right)=a_{film}\exp\left[-{\left(T-T_{film}\right)}^{2}/b_{film}^{2}\right]$$

where $T_{iron}$ and $T_{film}$ denote the MIT and the oxide film temperature, respectively.

The fitted curves are shown in Figure 5b, the Gaussian function of molten iron is marked in red, and the Gaussian function of oxide film is marked in blue. According to the Gaussian function obtained by fitting, we can obtain the temperature corresponding to the molten iron peak which is used to represent the MIT. Similarly, the temperature of oxide film can be obtained.

#### 2.2.3. Emissivity Evaluation

Emissivity is one of the key parameters of infrared thermal imagers, which has an important impact on the accuracy of infrared temperature measurement results [31,32]. When using the infrared thermal imager to measure temperature after the skimmer, it is necessary to estimate the emissivity of molten iron. However, emissivity evaluation is not a simple task because of the complex environment at the blast furnace site. Thus, this paper proposes a procedure to evaluate the molten iron emissivity at the blast furnace site.

Firstly, a gun with a thermocouple is used to measure the MIT after the skimmer, and its measurement result is recorded as a reference temperature. At the same time, the MIT at the same position is measured and recorded by the proposed measurement method. Then, the emissivity of the imager is continuously changed until the measurement results of the proposed method and the thermocouples are approximately equal, and the emissivity at this time is recorded as the effective emissivity of molten iron, and the emissivity is used as the set emissivity of the infrared thermal imager. It should be noted that the set emissivity is not necessarily the true emissivity of molten iron; thus, we call it the effective emissivity of molten iron, which is only suitable for temperature measurement of molten iron at the blast furnace site.

### 2.3. Temperature Measurement of Molten Iron at Taphole

The environment around the blast furnace taphole is worse than that around the skimmer. Although the aforementioned method can obtain the MIT after the skimmer, the MIT at the taphole is more sensitive to the thermal state in the blast furnace hearth. Therefore, if the relationship between the MIT at the taphole and the MIT after the skimmer can be established, the MIT at the taphole can be calculated in real time according to the MIT after the skimmer.

#### 2.3.1. Temperature Reduction Model

Generally, the MIT in the main pipeline is about 1500 °C, and the temperature of the surrounding objects and the environment is much lower than the MIT. Therefore, the molten iron suffers heat loss when it flows in the main pipeline. In [33], the heat loss of molten iron from the taphole to the skimmer was analyzed during the stable period of tapping, and the measurement results of thermocouples buried in trench walls were used as the input of the established temperature drop model to calculate the MIT at different positions in the main pipeline. Since these measurement results of the thermocouple have hysteresis, the calculated MIT also has a certain hysteresis. In order to obtain the MIT at the taphole in real time, this paper proposes a temperature reduction model, in which the MIT after the skimmer is used as model input, to calculate the MIT at the taphole during the stable period of tapping.

**Figure 6:**
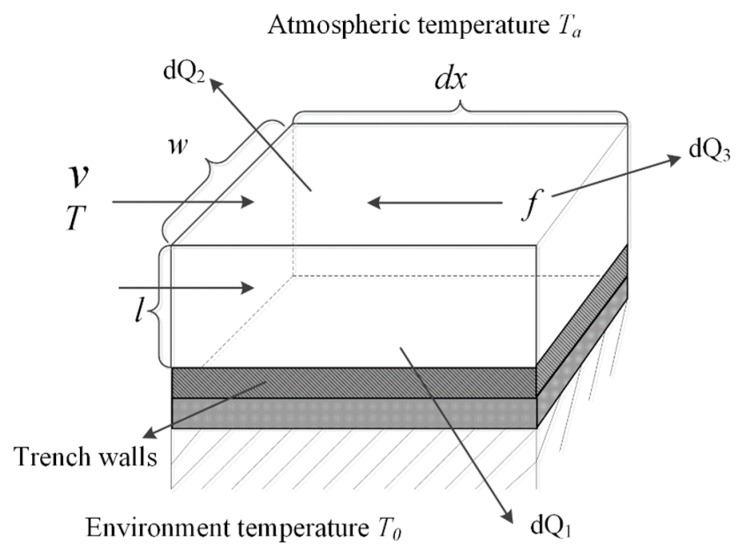
Structure of trench wall’s micro-element.

Firstly, based on the micro-element method, a heat loss model is established for the molten iron in the micro-element of the main pipeline, which is expressed as Equation (5). Figure 6 shows a structure of the micro-element of the main pipeline. There are three sources of the heat loss dQ of molten iron in the micro-element. The first source is the heat convection $dQ_{1}$ which consists of two parts: the heat convection loss between the bottom of trench wall and molten iron, and the heat convection loss between the lateral wall and molten iron. The loss of radiant heat and convective heat between molten iron and air is the second source $dQ_{2}$. And the third source is called the friction heat loss $dQ_{3}$, which is caused by the friction between molecules inside the molten iron.

(5) $$dQ=dQ_{1}+dQ_{2}+dQ_{3}$$

Based on Fourier’s law and Newton’s law of cooling, Equation (5) is expanded as:

(6) $$-GcdT=\lambda_{1}w\left(T-T_{0}\right)dx+2\lambda_{2}l\left(T-T_{0}\right)dx+\left(h_{R}+h_{C}\right)w\left(T-T_{a}\right)dx-gGidx$$

In Equation (6), $\lambda_{1}$ and $\lambda_{2}$ denote the coefficient of convective heat transfer between the bottom of the trench walls and molten iron and the coefficient of convective heat transfer coefficient between the lateral walls and molten iron, respectively. $h_{R}$ and $h_{C}$ denote the coefficient radiation heat transfer and the coefficient of convective heat transfer between molten iron and air, respectively. In the stable tapping period, the values of these coefficients are considered constants because the heat loss of the trench wall’s micro-element is stable. c and G denote the specific heat capacity and the mass flow, respectively.

Then, the temperature reduction model of molten iron in the main pipeline can be obtained by integrating the heat dissipation model in length, as shown in Equation (7).

(7) $$T_{L}=\alpha +(T_{t}-\alpha )e^{-\frac{L}{\beta }}$$

where $\alpha =\left(H_{1}T_{0}+H_{2}T_{a}\right){\left(H_{1}+H_{2}\right)}^{-1}$, $\beta =Gc{\left(H_{1}+H_{2}\right)}^{-1}$, $H_{1}=\lambda_{1}w+2\lambda_{2}l$, and $H_{2}=\left(h_{R}+h_{C}\right)w$.

In Equation (7), $T_{t}$ and $T_{L}$ denote the MIT at taphole and the MIT at a position L meters from the taphole, respectively.

At the BF casthouse of an ironmaking plant, the distance between the taphole and the skimmer is fixed, and it can be considered as a constant which is denoted as $L_{s}$. Thus, the relationship between the MIT at the taphole and the MIT after the skimmer can be obtained by transforming Equation (7).

(8) $$T_{t}=(T_{L_{s}}-\alpha )e^{\frac{L_{s}}{\beta }}+\alpha$$

where $T_{L_{s}}$ denotes the MIT after the skimmer.

Equation (8) is the temperature reduction model that characterizes the relationship between the MIT at the taphole and the MIT after the skimmer.

#### 2.3.2. Model Parameter Identification

Although the environmental temperature $T_{0}$, atmospheric temperature $T_{a}$ and mass flow G can be measured and considered as constants, the harsh environment makes it difficult to measure these heat transfer coefficients in different directions. As a result, the parameters α and β in Equation (8) cannot be determined. Since w and l are designed in advance, and they hardly change during the tapping process, the parameters $H_{1}$ and $H_{2}$ can be seen as constants according to the assumptions in [33]. Thus, the parameters α and β can be approximated as constants. Therefore, Equation (8) can be transformed into:

(9) $$T_{R}=kT_{L_{S}}+b$$

where $k=e^{\frac{L_{S}}{\beta }}$ and $b=\alpha \left(1-e^{\frac{L_{S}}{\beta }}\right)$.

In Equation (9), only parameters k and b are unknown. Thus, the least-squares method is utilized to identify the two unknown parameters [34]. The data, which is used for parameter identification, is measured at No.2 BF in an ironmaking plant and denoted as $\left(T_{ti},T_{L_{s}i}\right)$ (i=1,2,⋯,n). The $T_{ti}$ and $T_{L_{s}i}$ correspond to the MIT at the taphole and the MIT after the skimmer at different times.

According to Equation (9) and the collected data, the target function with the minimization of the square of error sums is established as follows:

(10) $$\min\;E\left(k,b\right)=\underset{k,b}{\min}\sum_{i=1}^{n}{\left(kT_{L_{s}i}+b-T_{ti}\right)}^{2}$$

Then, the parameter identification of the linear model can be obtained.

(11) $$\left\{ \begin{array}{l} k=\left(\sum_{i=1}^{n}T_{L_{s}i}T_{ti}-\frac{1}{n}\sum_{i=1}^{n}T_{L_{s}i}\sum_{i=1}^{n}T_{ti}\right){\left(\sum_{i=1}^{n}T_{L_{s}i}^{2}-\frac{1}{n}{\left(\sum_{i=1}^{n}T_{L_{s}i}\right)}^{2}\right)}^{-1}\\ b=\frac{1}{n}\sum_{i=1}^{n}T_{ti}-\left(\frac{1}{n}\sum_{i=1}^{n}T_{L_{s}i}\right)k \end{array}\right.$$

Finally, by taking the identified parameters into Equation (9), the MIT at the taphole can be calculated according to the MIT after the skimmer.

### 2.4. Procedure of Molten Iron Temperature Measurement

The implementation procedure of MIT detection is summarized as follows.

Step 1: Install the infrared thermal imager to capture the infrared images of molten iron flow after the skimmer.
Step 2: Process the infrared images to obtain the temperature information of the ROI2 which provides temperature data for the temperature mapping model.
Step 3: Detect the MIT after the skimmer according to temperature mapping model.
Step 4: Use the MIT after the skimmer as the input of the temperature reduction model, and obtain the MIT at the taphole.

## 3. Results and Discussion

To validate the proposed measurement method, industrial experiments were carried out at the No. 2 BF in an ironmaking plant of Liuzhou Steel Co. Ltd. which is the largest iron and steel enterprise in south-west China. The infrared thermal imager used to capture the molten iron flow after the skimmer is FLUKE TiX1000 which provides infrared images of 1024 × 768 pixels, and its measurement range is from 40 °C to 2000 °C. After a number of experimental correction after the skimmer, 0.56 is used as the effective emissivity of molten iron which is also the set emissivity of the imager. The infrared images of molten iron flow after the skimmer is captured every 10s by FLUKE TiX 1000, which means that a temperature value can be obtained every 10s. Thermocouples are simultaneously used to measure the MIT at the same measurement position, and their results are compared with those of the proposed method. Taking into account the safety of the on-site test and the measurement time limit of the thermocouples, it takes about 3 min to obtain one MIT by a thermocouple.

### 3.1. Results after Skimmer

**Figure 7:**
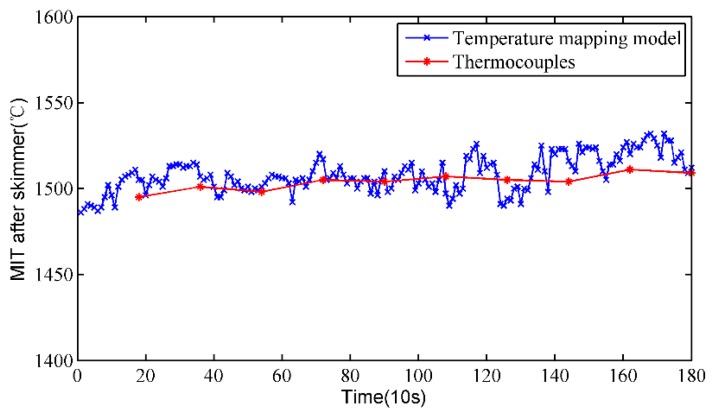
Results comparison between the temperature mapping model and thermocouples.

**Figure 8:**
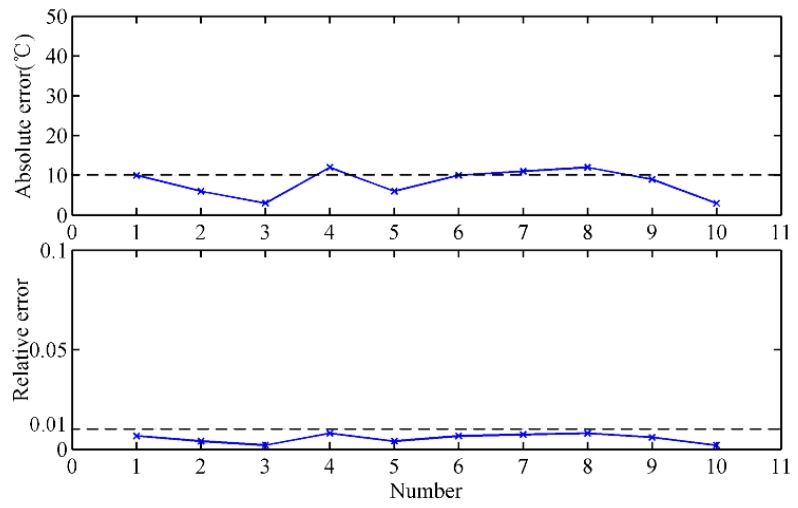
Measurement error analysis of the temperature mapping model.

Figure 7 shows the MIT after the skimmer measured by the temperature mapping model and thermocouples at the BF site in about 30 min. It can be seen that the measurement results of the infrared thermal imager are close to those obtained by thermocouples, and both these sets of data show essentially the same trend. Furthermore, the absolute error and the relative error are utilized to evaluate the accuracy and stability of the proposed method. Since there is no gold standard for temperature measurement of molten iron, the stable temperature measured by thermocouples is regarded as the reference temperature. The calculated absolute error and relative error are shown in Figure 8. The absolute error of the proposed method is almost smaller than 20 °C, and most of the relative error values are smaller than 1%. The measurement errors indicate that the results of the temperature mapping model are close to those of the thermocouples, and the temperature mapping model is reliable.

### 3.2. Results at Taphole

**Figure 9:**
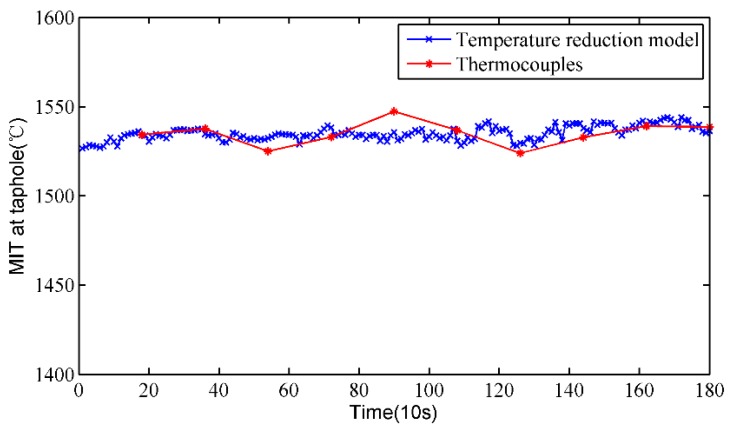
Results comparison between the temperature reduction model and thermocouples.

**Figure 10:**
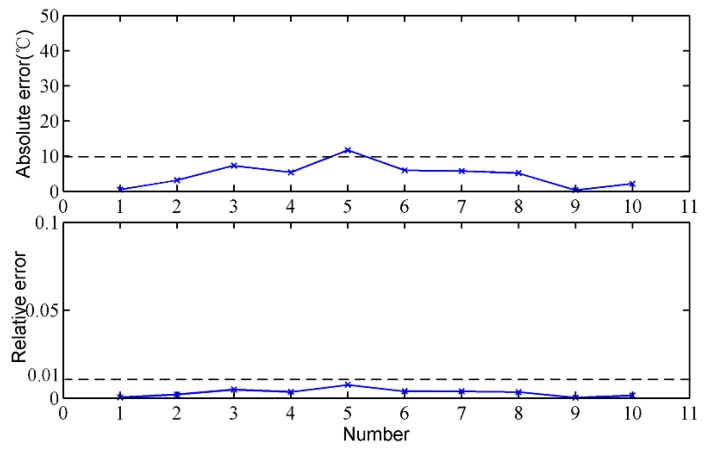
Measurement error analysis of the temperature reduction model.

To verify the rationality of the temperature reduction model, the MIT at the taphole is measured by thermocouples under the premise of ensuring safety, and the calculated results of the temperature drop model are compared with that of the thermocouples. Figure 9 shows the MIT at the taphole of the proposed method and the thermocouples. It can be clearly seen that the measurement results of the two methods are relatively close and the trends are basically the same. By the same token, we calculate the absolute and relative errors of the proposed method, which are shown in Figure 10. The absolute error of the proposed method is mostly less than 20 °C, and most of the relative error is less than 1%. The calculated measurement errors indicate that the calculated results of the temperature reduction model are close to those of the thermocouples, and the temperature reduction model is effective.

### 3.3. Discussion

Compared with thermocouples, the proposed method requires little manual intervention after its installation and can be operated safely, and BF operators do not have to face a certain danger when they are measuring near the high-temperature molten iron. Generally, the proposed methods can measure the molten iron temperature after the skimmer and at the taphole continuously, and the measurement results basically satisfy the requirement of BF operators. It should be pointed that the proposed measurement method sometimes still has measurement errors. In future research, we will manage to improve the proposed method to reduce the measurement errors.

## 4. Conclusions

The molten iron temperature of a blast furnace is an important parameter for monitoring molten iron quality and operating the blast furnace. In order to realize continuous temperature measurement of blast furnace molten iron, this paper proposes a measurement method based on infrared thermography and a temperature reduction model. The infrared thermal imager was applied to capture the infrared images of molten iron flow after the skimmer, and we established a temperature mapping model to continuously detect the molten iron temperature after the skimmer. Furthermore, the relationship between the molten iron temperature at the taphole and skimmer was described by a temperature reduction model in which the molten iron temperature after the skimmer was used as the input to obtain the molten iron temperature at the taphole.

Experimental results at No. 2 BF in an ironmaking plant indicate that the proposed method is capable of realizing continuous temperature measurement for molten iron at the taphole and skimmer. Moreover, it may provide inspiration for measuring the temperature of other molten metals.

## Figures and Tables

**Table 1:** Characteristic parameters of the infrared thermal imager.

Device	Characteristic Parameters	
Infrared thermal imager	Manufacturer and model	FLUKE TiX1000
	Measurement range	−40 °C–2000 °C
	Measurement accuracy	±1.5 °C
	Pixel resolution	1024 × 768
	Field of view	32.4° × 24.7°
	Operating temperature	−25 °C–55 °C
	Spectral range	7.5 μm–14 μm

**Table 2:** Emissivity of molten iron and oxide film.

Material	Emissivity
Molten iron	0.2–0.4
Oxide film	0.6–0.9
